# Modeling of HIV/AIDS dynamic evolution using non-homogeneous semi-markov process

**DOI:** 10.1186/2193-1801-3-537

**Published:** 2014-09-17

**Authors:** Zelalem Getahun Dessie

**Affiliations:** Department of Statistics, College of Science, Bahir Dar University, Bahir Dar, Ethiopia

**Keywords:** Disease progression, Non-homogeneous semi-Markov process, HIV/AIDS

## Abstract

The purpose of this study is to model the progression of HIV/AIDS disease of an individual patient under ART follow-up using non-homogeneous semi-Markov processes. The model focuses on the patient’s age as a relevant factor to forecast the transitions among the different levels of seriousness of the disease. A sample of 1456 patients was taken from a hospital record at Amhara Referral Hospitals, Amhara Region, Ethiopia, who were under ART follow up from June 2006 to August 2013. The states of disease progression adopted in the model were defined based on of the following CD4 cell counts: >500 cells/mm^3^ (SI); 349 to 500 cells/mm^3^ (SII); 199 to 350 cells/mm^3^(SIII); ≤200 cells/mm^3^ (SIV); and death (D). The first four states are referred as living states. The probability that an HIV/AIDS patient with any one of the living states will transition to the death state is greater with increasing age, irrespective of the current state and age of the patient. More generally, the probability of dying decreases with increasing CD4 counts over time. For an HIV/AIDS patient in a specific state of the disease, the probability of remaining in the same state decreases with increasing age. Within the living states, the results show that the probability of being in a better state is non-zero, but less than the probability of being in a worse state for all ages. A reliability analysis also revealed that the survival probabilities are all declining over time. Computed conditional probabilities show differential subject response that depends on the age of the patient. The dynamic nature of AIDS progression is confirmed with particular findings that patients are more likely to be in a worse state than a better one unless interventions are made. Our findings suggest that ongoing ART treatment services could be provided more effectively with careful consideration of the recent disease status of patients.

## Introduction

The rate of spread of the HIV/AIDS epidemic has reached a shocking level. On a global scale, the HIV epidemic has stabilized, although with unacceptably high levels of new HIV infections and AIDS deaths (United Nations Programme on HIV/AIDS UNAIDS 2013). The hallmark of the HIV infection is the progressive depletion of a class of lymphocytes named CD4+ which plays a pivotal regulatory role in the immune response to infections and tumors. The immune suppression resulting from the CD4+ decline leads to high susceptibility to opportunistic infections and possibly unusual tumors (Anderson et al. 1986; Satten and Longini 1999; Dessie 2014b). Infection by the human immunodeficiency virus (HIV) gradually evolves to the acquired immune deficiency syndrome (AIDS), and AIDS evolves to death if not handled carefully. One may consider this progression of HIV infection to AIDS and then to death as a stochastic process, by splitting the progression into various states of the disease based on the immunological indicators, namely CD4+ count, including death as one state (Janssen and Manca 2001).

Many HIV/AIDS patients are being treated with drug antiretroviral therapy (ART) that has been found to reduce mortality and improve quality of life of the patients. The effect, however, varies from country to country (Braitstein et al. 2006). Egger (2007) indicated that there are several predictors of mortality for HIV/AIDS patients who are undergoing treatment on ART, including CD4 count, viral load, total lymphocytes, body mass index and adherence.

Recent studies have shown that the predicted probability of a patient changing his or her status given his or her current status allows for improved treatment of the HIV/AIDS (Goshu and Dessie 2013). Since the discovery of HIV/AIDS, numerous mathematical models Corradi et al. (2004), Ouhbi and Limnios (1999) and Blasi and Manca (2004) have been developed to describe infectious disease transmission dynamics, assess the impact of interventions on public health or forecast the future of epidemics. Among recent papers in biomedicine, Giuseppe et al. (2007) and Dessie (2014a) analyzed HIV/AIDS dynamic evolution as defined by CD4 levels from a macroscopic point of view by means of homogeneous semi-Markov processes. Other relevant publications include (Davidov and Zelen (2000); Limnios and Oprisan (2000); and Jannsen and Manca (1997)).

Non-homogeneous semi-Markov Processes (NHSMP) were defined the first time in Iosifescu-Manu (1972). The discrete time NHSMP were defined in Janssen and De Dominicis (1984). Several authors have discussed the use of non homogeneous semi-Markov in analysis of health data, including (but not limited to) (D'Amico et al. (2009); Mathieu et al. (2007) and Andersen et al. (1991)). Another approach to NHSMPs was presented in Vassiliou and Papadopoulou (1992).

In this paper, the author proposes the application of the non-homogeneous Markov process to study the evolution of HIV/AIDS. The consideration of the non-homogeneity allows the researcher to take into account not only the length of the period of the HIV patients as well as the randomness in the different states in which the infection can evolve. This approach also allows one to differentiate patient's age according to the starting time. The author also presents the results of modelling of the progression of HIV/AIDS with a focus on the patient’s age as a relevant factor to predict the future clinical state and survival probability of a patient.

## Methods

### Ethics Statement

This investigation was conducted according to the principles expressed in the Declaration of Bahir Dar University, Ethiopia. It was approved by the research ethics committee at the University of Bahir Dar and all participants who agreed to participate in this study signed a consent form.

### Data and model descriptions

The target population for this study was patients under the follow up of ART at Amhara Referral Hospitals, Amhara Region, Ethiopia from June 2006 to August 2013. Multistage sampling was used to select study subjects. The calculated sample size was proportionally allocated to Eastern (n = 482) and Western (n = 874) areas respectively. The sampling frame consists of 65000 HIV/AIDS patients who have visited the hospitals since the initiation of ART. The study may consider all HIV infected patients under ART whose age is ≥15 years regardless of their treatment category during the study period in Amhara Referral Hospitals, Amhara Region, Ethiopia.

**Figure 1:**
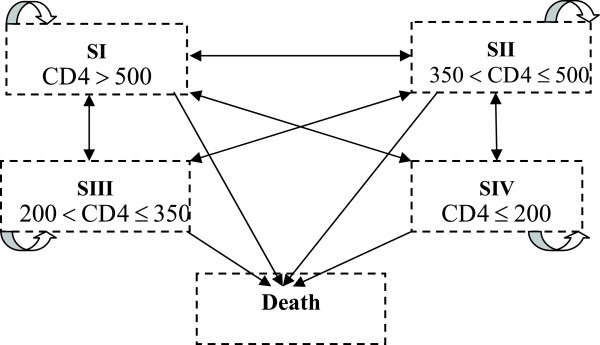
**Communication between the states of the process.**

The states of disease progression adopted in the multi-state model were defined based on the following CD4 cell counts as in Giuseppe et al. (2007): >500 cells/mm^3^ (SI); 349 to 500 cells/mm^3^ (SII); 199 to 350 cells/mm^3^(SIII); ≤200 cells/mm^3^ (SIV); and death (D). The death state is considered to be an absorbing state, meaning that once a patient is in the death state she/he will remain in that state forever. The time elapse between state transitions was determined using the difference (in years) between the dates of CD4 tests.In Figure 1 the graph model is displayed. It shows all the immunological states a HIV infected patient can go into. All the states apart from the death state are inter-related, and also improvements (moving into a higher CD4 cell count state) are considered. It is also possible that an examination will show that the patient’s state has not changed.

### Non-homogenous semi-Markov processes

In this part, the non-homogeneous semi-Markov model is described using the notation of D’Amico et al. (2010).

On a complete probability space the author define three random variables. Let *J*_*n*_ : *Ω* → *S* be the stochastic process with state space *S* = {*S*_1_, *S*_2_ … *S*_*m*_}, *T*_*n*_ : *Ω* → *N* be the time of the n^th^ transition and *A*_*n*_ : *Ω* → *N* be the age of the patient when she/he had the n^th^ transition, with *Ω* domain of the process and *N* set natural numbers. Here the time is a random variable.

The kernel Q = [Q_ij_(s,t)] associated with the process and the transition matrix P_ij_(s,t) of the embedded Markov chain is defined as follows:
2.12.2

Define the probability of making next transition before time *t* being in state *i* at time *s* with age a + *s* is given by
2.3

The distribution of waiting time in each state i, given that the state j is subsequently occupied is
2.4

For any non-homogeneous semi-Markov process {^*a*^*Z*(*t*) = *J*_*aN*(*t*)_, *t* ∈ *N*}, the transition probabilities are given by (5) for which the solutions should be obtained using the progression (6).
2.52.6

Here *δ*_*ij*_ represents the Kronecker delta. An approximate solution of (6) can be obtained using the general numerical integration formula given in Janssen and Manca (2001). In the same paper, they proved that the numerical solution of the process converges to the discrete time NHSMP described as an infinite countable linear system:
2.7

Where the term  represents the probability of entering in any state l at any time *τ* with next transition given the entrance with last transition in state *i* at time *s* with age *a* + *s*. and
2.8

In matrix form, Equation (8) becomes:
2.9

The fact that the matrix ***Φ***(*s*, *t*) is stochastic is already proved in Janssen and Manca (2001). The following algorithm with suggested matrix form is used for solving the evolution Equation (9):
2.10

The variables involved are the following:

*m* = number of states of SMP.

***T*** = number of periods to be examined for the transient analysis of SMP.

***P***_(s)_ = matrix of order m of the embedded markov chain in SMP.

***G*** = square upper-triangular block matrix of order T + 1 whose blocks are of order m.

***Q*** =It represents the kernel of SMP.

***Φ*** =square upper-triangular block matrix of order T + 1 whose blocks are of order m.

***D*** =square upper-block matrix of order T + 1whose blocks are of order m.

**V** = square lower-triangular block matrix of order T + 1 whose blocks are of order m.

**H** = block vector of order T + 1 the block of which are the diagonal square matrix of order m. the diagonal element of each block t is .

Given an epoch s, T, matrices *G*^*T*^ and P, the algorithm solves the linear system (10) for the unknown matrix *Φ*(*s*, *T*) by means of a purely iterative procedure. The algorithm is:

(0) Read the inputs: *m*, *s*, ***T***, ***P***(***s***), ***G***(***s***)
(1) Construct: ***Q***(***s***, ***T***), **V**(**s**, **T**), ***D***(***s***, ***T***)
(2) Given **Φ**_(*s*,*s*)_ = *D*_(*s*,*s*)_, solve for **Φ**(*s*, *T*)
(3) Print the results, **Φ**(***s***, ***T***), ***Q***(***s***, ***T***)

In the above (•) represent the usual row column matrix product (∗) stands for element by element product and (**1**) is the m-component’s sum vector.

In predicting the survival probability of a patient, let us first group the states of the process into two sets A and B, where A contains all “living" states in which the patient is alive and set B contains all “bad" states in which the patient is not alive. Then the probability that the patient will survive up to time *t* given the entrance into degree of illness *i* at time *s* with an age of *a* + *s* is given as:
2.11

The algorithm is programmed in the R statistical software version 2.6.2.

## Results and discussion

**Figure 2:**
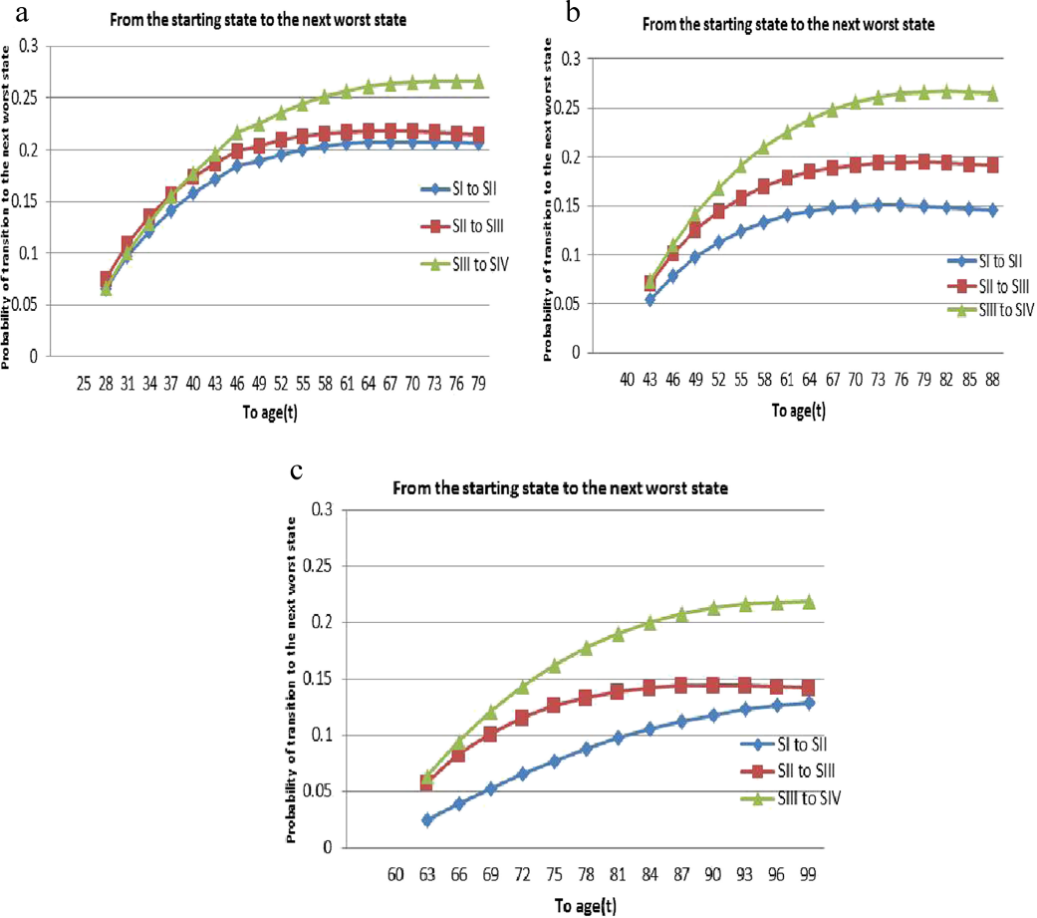
**The conditional probability that an HIV/AIDS patient to get “worse" state up to the age “t” if he/she entered state** ***i*** **∈** **{** ***SI*** **,** ***SII*** **,** ***SIII*** **,** ***SIV*** **} at: (a) age 25 (b) age 40; (c) age 60.**

**Figure 3:**
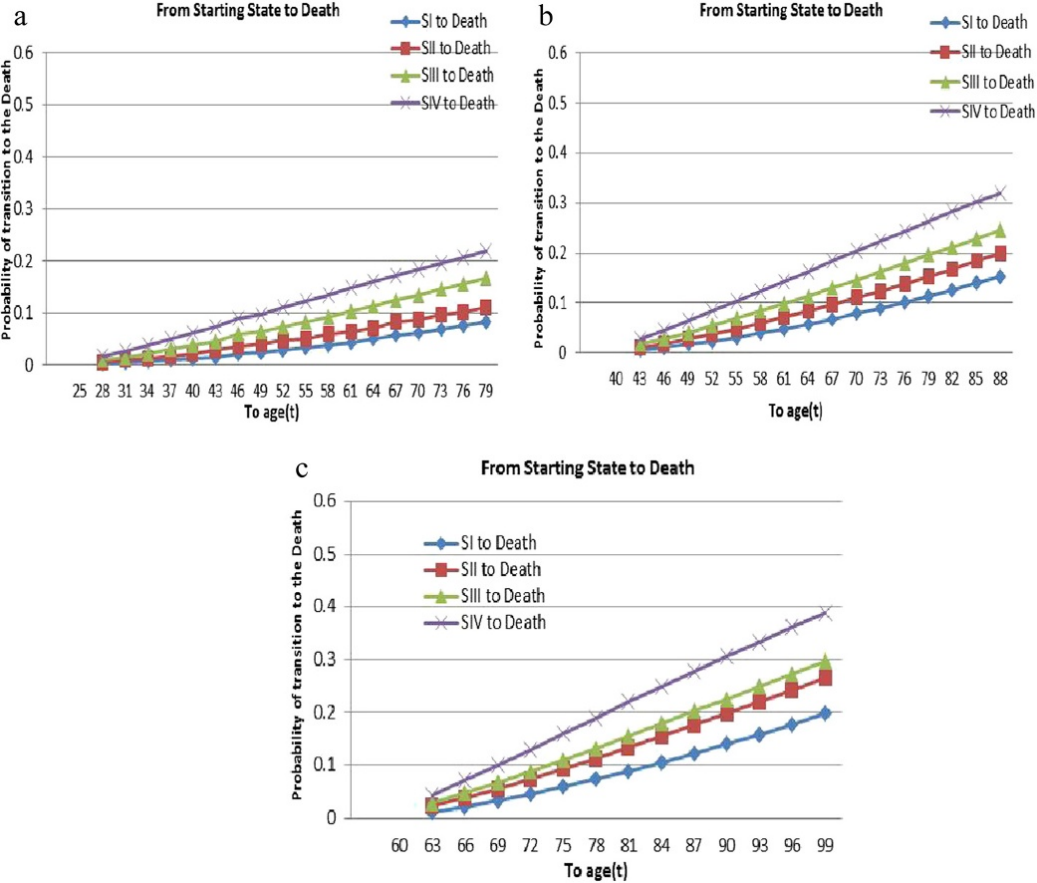
**The conditional probability that an HIV/AIDS patient to get “death" state up to the age “t” if he/she entered state** ***i*** ∈ **{** ***SI*** **,** ***SII*** **,** ***SIII*** **,** ***SIV*** **}** **at: (a) age 25 (b) age 40; (c) age 60.**

**Figure 4:**
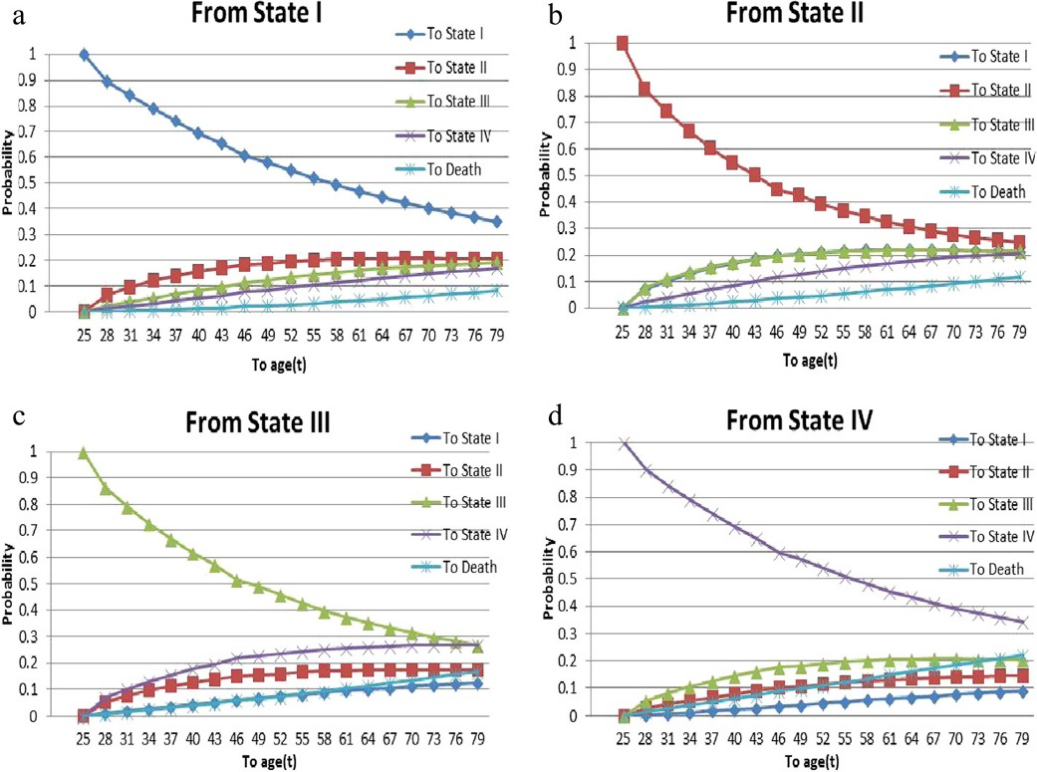
**The conditional probability that an HIV/AIDS patient to get state** ***j*** ∈ **{** ***SI*** **,** ***SII*** **,** ***SIII*** **,** ***SIV*** **,** ***D*** **}** **at age “t” if he/she entered. (a)** State I at age 25, **(b)** State II at age 25, **(c)** State III at age 25, **(d)** State IV at age 25.

**Figure 5:**
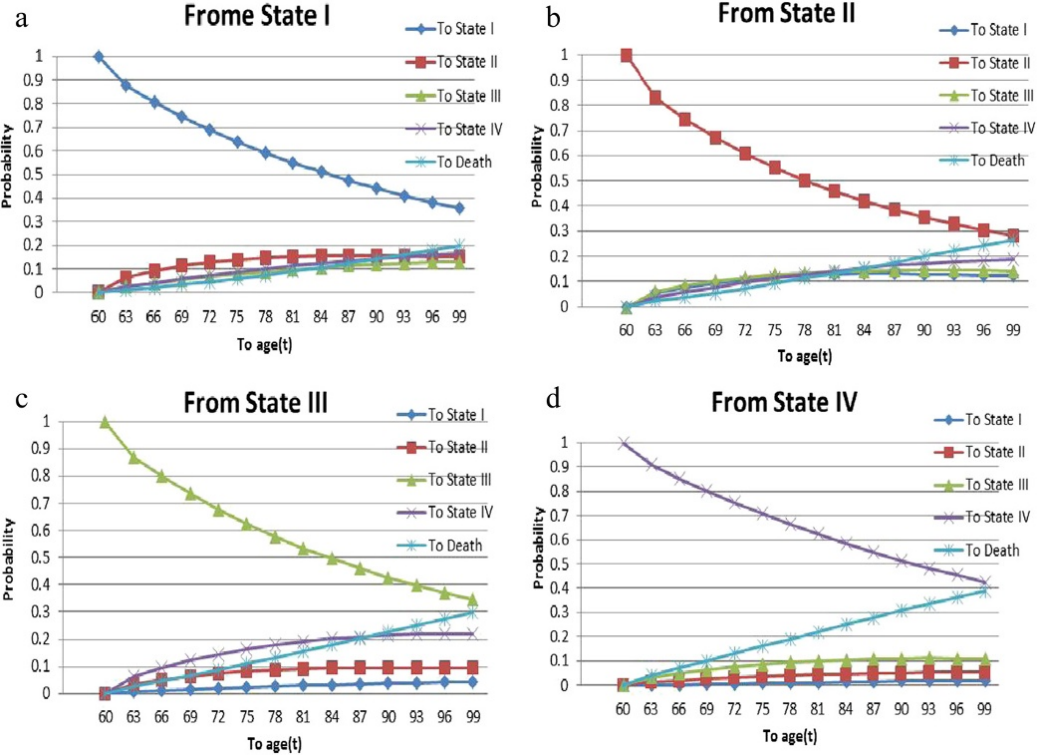
**The conditional probability that an HIV/AIDS patient to get state** ***j*** ∈ **{** ***SI*** **,** ***SII*** **,** ***SIII*** **,** ***SIV*** **,** ***D*** **} at age “t” if he/she entered. (a)** State I at age 60, **(b)** State II at age 60, **(c)** State III at age 60, **(d)** State IV at age 60.

**Figure 6:**
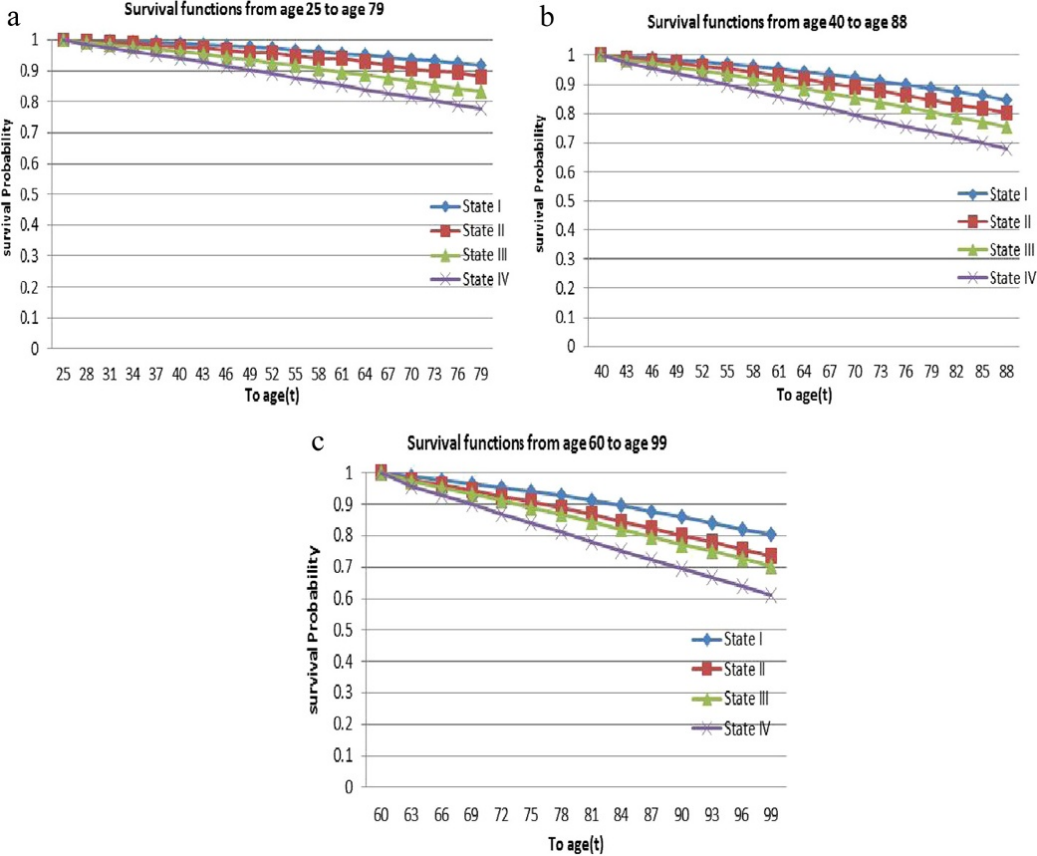
**Survival functions. (a)** From age 25 to age 79; **(b)** From age 40 to age 88; **(c)** From age 60 to age 99.

Results of modelling is displayed in Figures 2, 3, 4, 5 and 6.

First, transitions within the “good” states are considered. The conditional probability that an HIV/AIDS patient to get “worse" state at age “t” if he/she entered state *i* ∈ {*SI*, *SII*, *SIII*, *SIV*} at age 25, 40 and 60 are displayed in Figure 2(a), (b) and (c), respectively. Such progressions are from SI to SII, SII to SIII and SIII to SIV. Each plot is parabolic curve with optimal/peak points in the age-probability axis. The peaks may indicate there is time when a patient will be at highest risk of being at worse state. Moreover, the transition probability from state one to state two is the lowest as compared to the others for all ages. It is interesting to observe that, within the good states, the transition probabilities are different when the age changes.

Second, transitions to the “bad" or “death” states are considered. The conditional probability that an HIV/AIDS patient transitioned to “bad” or “death” state up to the age “t” if he/she entered state *i* ∈ {*SI*, *SII*, *SIII*, *SIV*} at age 25, 40 and 60 are displayed in Figure 3(a), (b) and (c), respectively. Such progressions are from SI to D, SII to D, SIII to D and SIV to D. The probability of dying up to age 80 is 0.09 for the patient who entered state I at age 25, 0.11 for one who entered state one at age 40, and 0.10 for one who entered state one at age 60. Similarly, the probability of dying up to age 80 is 0.11 for the patient who entered state two at age 25, 0.15 for one who entered state two at age 40 and 0.15 for one who entered state two at age 60. Each plot is an increasing parabolic curve over time (age) with no optimal/peak point. Moreover, a patient who is in the fourth state has the highest probability of dying, while a patient who is in the first state has the lowest probability of dying throughout the time for all ages.

Third, the conditional probability of a patient making changes in disease states given his/her current status is computed and displayed in Figures 4 and 5. The results show that the probability that an HIV/AIDS patient transitions *j* ∈ {*SI*, *SII*, *SIII*, *SIV*, *D*} at age “t” if he/she entered state *i* ∈ {*SI*, *SII*, *SIII*, *SIV*} at age 25 and 60, respectively. The results plotted can be interpreted as follows. For an HIV/AIDS patient in a specific state of the disease, the probability of remaining in the same state decreases with increasing ages. With the good or alive states, the results show that probability of being in a better state is non-zero, but less than the probability of being in worst states for all ages. That is, for a patient there is more likely to be in worse state than to be in better one, relatively speaking.

Finally, the probabilities to survive up to age “t” given that the patient entered state *i* ∈ {*SI*, *SII*, *SIII*, *SIV*} at age 25, 40 and 60 are displayed in Figure 6(a), (b) and (c), respectively. The survival probability that a patient who entered state one at age 25 will be alive until age 80 is about 0.92, 0.89 for one who entered state one at age 40 and 0.91 for one who entered state one at age 60. Similarly, the survival probability that a patient who entered state two at age 25 will be alive until age 80 is about 0.88, 0.85 for one who entered state two at age 40 and 0.87 for one who entered state two at age 60. Thus patient in the first state have better chance of survival than patients in other states for all ages. Other results in Figure 6 also reveal that the survival probabilities are all decreasing with increasing ages.

Markov models of the natural history of HIV, especially those based on CD4 cell counts, play a central role in AIDS modelling. They have been used to describe the natural history of HIV infection (Longini et al. 1991), to predict the stage-specific course of the HIV epidemic in the USA (Longini et al. 1992), and to evaluate the effect of covariate such as therapy on stage-specific progression rate (Foucher et al. 2005). The non-homogeneous semi- Markov model proposed by Hoem (1972), further studied by Iosifescu-Manu (1972), and applied by Janssen and De Dominics (1984) and De Dominicis and Manca (1984) for studying the effect of disease indicators on survival was used in this paper to model the progression of HIV/AIDS with a focus on patient’s age.

We applied non homogeneous semi-Markov processes to assess the dynamic evolution of the Human Immunodeficiency Virus Infection, as defined by CD4+ level. The large number of results obtainable by the model included the probabilities of an infected patient’s survival taking into account the patient’s age. Indeed, by means of the non-homogeneous model it is possible to study the dynamic evolution of the infection differentiated according to the patient’s age. Moreover the age of the infected patients significantly diversifies the disease evolution. This results presented above is similar to the result obtained in previous studies (D'Amico et al. 2011; Jackson *et al*. 2003).

## Conclusions and recommendations

The semi-Markov process model is applied to capture the AIDS dynamic progression of a patient. The model considers the length of the period of the HIV patients, the randomness in the different states in which the infection can evolve, and patient's age according to the starting time. The following can be concluded from this study.

The probability that an HIV/AIDS patient with any one of the good states will transition to the death state is increasing with greater age, irrespective of the current state and age of the patient. More generally, the probability of dying decreases with increasing CD4 counts over time. For an HIV/AIDS patient in a specific state of the disease, the probability of remaining in the same state decreases with increasing age. Within the good states, the results show that the probability of being in a better state is non-zero, but less than the probability of being in a worse state for all ages. At any time of the process, there is more likely to be in worse state than to be in better one. The reliability analysis indicates that the survival probabilities are all decreasing with increasing ages.

In general, the survival probability of an HIV/AIDS patient depends on his/her current state of the disease in such a way that lower CD4 counts are associated with higher risk of transitioning to a worse health state or death state. The dynamic nature of the AIDS progression is confirmed with particular findings that there is more likely to be in worse state than better one unless interventions are made. It is recommendable to keep up the ongoing ART treatment services in most effective ways with the careful considerations of recent disease status of patients.
